# Supporting employability through sport: what kind of training?

**DOI:** 10.3389/fspor.2023.1154533

**Published:** 2023-06-23

**Authors:** Caterina Gozzoli, Martina Palumbo, Edgardo Zanoli

**Affiliations:** ^1^ Department of Psychology, Cattolica University, Milan, Italy; ^2^ Agostino Gemelli High School of Psychology (ASAG), Cattolica University, Milan, Italy

**Keywords:** coach training, SfE programmes, sport for employability (SfE), sport for development (SfD), training process assessment

## Abstract

Training, as a core device in the projects that use sport to increase employability, is today a much-cited element in papers in the sector. However, there seems to be little research that specifically delves into training processes. This contribution analyses the state of the art on the subject, focusing on the characteristics of the training courses mentioned in literature, highlighting some recurring critical issues. A proposal, which considers the limitations discussed above, is formulated as a result of this analysis. More specifically, we introduce, as a contribution to the debate, a training model for team sports coaches developed within the EU Erasmus + sport project SBSMED. Theoretical assumptions, methodology, contents and assessment methods of training effectiveness will be explained, highlighting valuable elements and the open issues that emerge from this experience.

## Introduction

### Sport and employability, the specific weight of training

For nearly twenty years now, sport has been considered a useful experience for the development of skills transferable to different areas of life (1–14), with a recent emphasis on the specific pairing of Sport and Employability (15–22).

The Work Plan for Sport 2017–2020 of the EU (European Union 2017) also mentions, among its priorities, the development of sport as a means to contrast unemployment, activating various policies to this end, and funding several programmes (Team up for NEET; SK4YS- Skills for Youth through Sport; Sport4employability; SCORES- developing skills and competencies resulting in employability through sport).

In this active debate, training is mentioned in almost all contributions as a crucial element of this effort. Nevertheless, training proposals are rarely discussed. It is often unclear, as several authors point out, what type of training, what specific characteristics it must have, and on what basis it is supposed to truly foster employability (16, 19, 23–25). By analyzing the literature on the topic in depth, we will try to highlight the most critical aspects which, in our opinion, should not be neglected in new studies and experiences.

The analysis reveals that relation between employability through sport, and the training programs on the theme, are often structured through implicit or imposed assumptions, which are methodologically weak, rather than through real strategies of action, with clear expected results (19); we will now highlight some of these *critical aspects*, which are useful to focus on the most relevant weaknesses in the state of the art.

A first critical element concerns precisely the *lack of clarification of the theoretical, methodological and contextual assumptions* that guide the design of training courses (and often, in general, also the entire design intentionality).

It seems useful in this regard to consider that most of these are top-down proposals, based on an often-unjustified selection of certain skills (among the many available), deemed useful in the labour market or more generally in social life, submitted in a transmissive and heterogeneous way to the various recipients (16, 26).

A dialogic, bottom-up approach, co-designed with practitioners, capable of highlighting the specific desirable skills, making the training offer situated and shared, is seldom submitted (25, 27, 28).

The characteristics of the recipients (which mainly focus on structural biographical data), their previous training experiences and the new needs on which the proposals are based are rarely valorised. Recruitment and selection of recipients are thus often the result of *a priori* defined or weakly justifiable processes (19, 29). This results is a generic training content, certainly useful in terms of increasing knowledge of the topic, but not incisive in terms of practical effects (30).

More specifically, concerning the *type of competences promoted in training courses* aimed at the employability of recipients, comparing the competence models developed in over 300 European sports projects (3), and the most recent systematisations of some users (19), it can be noted that the most in demand are: self-management, time management, resilience, generic social skills, personal effectiveness, self-esteem, teamwork, problem-solving.

Several issues need to be taken into consideration, in addition to the already highlighted explanation of why these skills were chosen: there appears to be an ambiguity between employability skills, life skills, soft skills, borrowing an imprecise equivalence between employability and life skills or between employability and soft skills (10). These questions point to the need to make explicit which idea of employability is being referred to and what kind of use of sport as a tool is being implied.

There seems to be an expectation that participation in sport and related activities inherently produces developmental outcomes (31), which are automatically transferable to other life contexts (18, 32, 33). The *expectation of an intrinsic transferability*, from the sporting sphere to the labour market, without presupposing any personal elaboration or reinterpretation, produces the skill mismatch, i.e., the gap between the skills required by the labour market and those developed during training programmes through sport (14, 34). It is possible to observe a *paradoxical exclusion of certain key targets,* which could facilitate awareness and transferability of learning, for the implementation of training proposals useful to young athletes, specifically coaches.

Indeed, another critical issue to highlight concerns the targets to which these training courses are most frequently addressed. Literature shows that the recipients are mostly young, elite athletes engaged in formal training courses, especially university students, with the aim of developing dual career paths and reconciling sport and education (17, 35–49). Other programmes, on the other hand, address two main targets, whose professional vulnerability is recognized as an urgent social risk; we refer specifically to Neets (Not in Education, Employment or Training) and migrants in different Western countries, in particular women, although they are more often involved in more generic social inclusion programs (14, 16, 19, 21, 22, 29, 34, 50–54).

Therefore, even though coaches are unanimously recognised as figures to be included in training courses, they hardly appear as protagonists in training proposals, thus becoming "great absentees". The *quality of the sports experience*, mediated by the coach teaching a particular sport, does not, therefore, seem to be a central theme. The exclusion of coaches from training projects and their instrumental inclusion in programs aimed at the main target groups (youths, NEETs and migrants), however, introduces the additional implicit assumption that coaches are always considered to be competent and knowledgeable (on methods of teaching sports, on issues of inclusion and employability, on knowing how to adapt them to different age groups) (55–57).

As we will cover in more detail below, a fundamental issue seems to be completely overlooked, namely that the practices put in place by coaches to support the employability of athletes should primarily aim at improving sports performance. The improvement of performance, therefore, should not be a separate and ancillary item, but the main objective of the training. Coaches and athletes should share the intent to improve in a specific sport. This is the only way in which the coach's function is not distorted and training programmes aimed at employability can contribute to real sport-based professional growth.

This last consideration appears to be connected to a further critical aspect, which concerns the *relationship between educational programmes for employability and sport*. Referring to Coalter's (26) distinction, two types of programmes are prevalent in training. The first, known as *plus-sport*, in which sport is a peripheral activity, an expedient used to strategically reach people who live on the margins or are excluded from society. The second, named *sport-plus*, in which the sporting environment is strategically manipulated to convey certain values or promote specific changes, integrating additional activities, e.g., workshops; these are aimed at developing awareness or stimulating social changes, with the risk, however, of subtracting sport from sport, minimising its competitive dimension and downplaying its authenticity. This implies a flattening of the sporting dimension, to the detriment of *sport-based* proposals, in which sport, with its intrinsic training potential, remains the focus of the intervention and in which the knowledge and teaching skills of the specific sport are the main competence. The choice of sport to develop skills is usually approached in a non-specific manner, without considering that some sports are more suitable for developing certain skills and less suitable for developing others, and overlooking the fact that the ways in which these skills are developed are also sport-specific.

A final critical issue that appears relevant concerns the evaluation of training processes, a *stage that is generally omitted or implemented only after the program is completed* [Cfr. (58)], as well as the explication of expected outcomes (59). It would be useful to assess the program's adherence to the project's objectives and indicators even before and during its implementation, considering possible reshaping and revisions on an ongoing basis, resulting in continuous and participatory evaluation of the training pathway. This would, consequently, mean adopting a participatory approach to evaluation and, more generally, to the design of the training course, promoting them as a starting point for multiple transformative learning processes (51, 60–63). It would also be desirable to understand the *impact* of the interventions implemented following the training completion, monitoring their *effectiveness and legacy* (64).

These recurring critical elements, which characterise the topic of training, promoted and implemented in projects and programmes that aim to increase employability through sport, are the result of a reasoned synthesis, derived from an in-depth and extensive analysis of the literature on the topic. We interrogated the existing literature by means of several specific and extensive search queries, which have returned scarce and fragmented results. For the sake of completeness, we report the outcome of the literature exploration carried out in the Appendix (Appendix^1^), where it is possible to observe the transparent research process that led to the present assertions, in order to be able to replicate or verify their consistency. In the state of the art, studies capable of providing a broad, explicit and in-depth overview of the use of training for the strategic purposes of increasing or supporting employability through sport seem to be absent.

The choice of using a critical-thematic synthesis (65), therefore appears more consistent at this stage of literature development, rather than conducting a systematic review on the topic. It is, therefore, considered premature to attempt a comprehensive systematic review on the basis of the existing literature, which is still scarce and in the making. We specify that the systematisation appears premature not because the literature is meagre and multifaceted (66), but because it is devoid of punctual considerations on the training intentionality, limiting itself to describing what has been done and not the contextual, theoretical, methodological configurations that have produced targeted impacts of success or failure, in agreement with what colleagues claim in authoritative and recent contributions on the subject (19, 25, 67).

A training course was outlined to accommodate these challenges, beginning with reflection on these critical issues, over a wide time frame and with the involvement of multiple European partners. The result is the SBSMED programme, we initially present the debate on the *fundamental constructs* that animate this training proposal, thus clarifying our theoretical positioning; subsequently, we present an *overview of the programme* structure, focusing on the development of the training modules, thus describing the practical precipitates of our theoretical positioning.

## The SBSMED programme

### The SBSMED goals: “making coaches aware of sport for employability”

The Skills By Sport for Med (SBSMED)^2^ programme is developed as part of the 3-year European project Erasmus + Sport; this programme was launched in January 2020 and is the result of a collaborative partnership between institutions in Portugal (ISCTE and Coaches Portugal), Italy (Catholic University and Okkam Srl), Qatar/England (International Center for Sport Security and Save the dream), Croatia (HAŠK Mladost), Greece (The International Olympic Truce Centre), Spain (Universitat de les Illes Balears) and Cyprus (Cyprus Sports Organisation). The project envisages sport as a vehicle for developing skills for the labour market, promoting employability. The aim is *to promote coaches' awareness of their role in supporting employability through the practice of sport in their athletes*. This objective is in line with the main priorities of EU policies, not only within the framework of sport, but also integration, immigration, development, security and international cooperation. The main target groups of the project are coaches and sports instructors who coach unemployed young people and immigrants, who can be supported by sport to integrate into the labour market.

### Core constructs and our positioning

To make our position in the debate explicit, we present the *core constructs* at stake, specifically delving into the relationship between *employability* and *sport,* and between *training* and *coaches*.

Employability is a complex and multidimensional construct that has assumed various facets over time.

Initially, employability was defined as the *possession* of a set of skills, totally ascribable to the individual dimension, to determine which abilities an individual should possess or develop to enter and remain in employment (68–71). Examples of such skills are time management, self-management, creativity, communication, reliability, problem-solving, teamwork (72, 73).

Subsequently, there is a shift to a notion of employability as a *position,* in which the work and social context are considered central. In particular, the number and type of job offers available, the specific skills required for that position, the competition, and the location of the job (74).

More recent perspectives, referred to in this contribution, conceive employability as a *process*, a composite psychosocial construct (75). Within this perspective, for instance, Fugate, Kinicki and Ashforth (76), identify three key dimensions of employability: personal adaptability, career identity, and human and social capital, i.e.,: "*A form of work-specific active adaptability that enables workers to identify and realise career opportunities"* (76).

In this sense, employability is configured as a construct intertwined with specific competences, animated by the perception of being capable, of doing things considered useful for oneself in the present, of feeling a good relational quality with reference figures, of being curious about what surrounds us. This is why the coach becomes a key figure to support this process within the sporting practice.

Regarding the relationship between employability and sporting activity, on the other hand, we consider that the growing trend in the literature in recognising sport as a tool for increasing employability (16) is due to an increased focus on the benefits, rather than a critical examination of the critical and deficient issues that this relationship presents (77). While scientific and political literature emphasize the sports tool and its ability to transmit certain skills to increase employability, it is also noted that the constructs are used imprecisely, and there is a lack of studies that evaluate in-depth whether and how sports can contribute to the promotion of employability (23). It emerges, in fact, that in many circumstances, participation in sport is a necessary, but not sufficient, condition for achieving personal and social outcomes (16). At the same time, the concept of employability continues to be used in a very large number of contexts and with different meanings (71, 78, 79). In addition, there is a widespread gap regarding explicit training strategies to be deployed in order to foster transferability from the sporting field to the working field; there appears to be a lack of an effective underlying rationale that has been adequately designed and tested (80). A very important aspect is that although coaches can play a key role in supporting this process, they are often the great absentees from training proposals (81).

In many studies, the coach is considered the most important figure in creating an adequate motivational climate and in proposing structured activities that enable young people to achieve their developmental goals (4, 31, 82, 83). To delineate the training pathway, we started by asking: *what contexts do coaches attend? What is the nature of their knowledge? What characteristics and training needs characterise them?* (60, 84). Practical experience as players and observation of colleagues seems to be the primary sources of coaches' knowledge, in terms of "informal" knowledge (85–89). Coaching refers to what Bourdieu (90) called "*habitus*", a set of internalised patterns through which people perceive, produce and evaluate their practices. Talking about the habitus of coaches means emphasising that, what they do on a daily basis has strong links to their history, the social positions they occupy and the continuous redefinition of their practices in their interactions with people and the environment. For this reason, it seems fundamental for their learning process to activate reflection on their experience (91–93). Conversely, if accumulated experience does not become the object of deconstruction and re-signification and remains the basis on which to layer additional content, it risks becoming more of a disadvantage than a resource. We found this to be the reason why formal programmes offered by federations and various institutions often seem to have a minimal impact on the professional activity of coaches compared to informal learning contexts (e.g., informal mentoring received from other coaches, coaching experience and interaction with other colleagues and one's own athletes) (85, 94). A further limitation of coach training seems to be the division between technical and soft contents: for a long time, training proposals in the sports field have been characterized by technical content related to performance in a narrow sense, promoting a “technocratic rationality” of the role (85, 95). More recently, there have been proposals focused on soft skills, but separated from the technicality of training. In both cases, such programmes often seem to neglect the contextual and dynamic aspects of the coaching process, reinforcing the simplified representation of the coach (87, 96). Although coaches can undoubtedly be considered a reference figure within any sports program, focusing exclusively on them is simplistic and incomplete. It is important to address the issue of resources in a broader sense when designing programs, rigorously and punctually questioning which contexts, figures, and relationships impact the athlete's development and skills.

In the light of these reflections, what training proposal can be outlined to effectively support coaches in their role as trainers and promote the employability of young athletes? Our perspective is based on the constructivist perspective (97–100) which states that in the process of generating knowledge, reflection in action and on action is fundamental. Work experience is the privileged field on which to develop knowledge, as each participant has direct experience and brings prior knowledge on the topic of training (99, 101–103). The training process cannot therefore be conceived as linear and predetermined in detail *a priori*, but rather as a process open to emergences and continuous redesign; focused on subjectivity, reflective capacities of daily practices, and current knowledge and theories (5, 104, 105). To this end, what can be defined as “temporary training-research organizations” (an organization, a space/time environment aimed at training) are proposed, with a defined and limited duration (in time), with the aim of supporting individuals in learning through the group tool. This training approach, alternated and integrated into the ordinary professional activity of individuals, is based on a conception of learning characterized by four fundamental elements: 1) Continuous reflection on one's own experience (for example, professional history and practices, relationship between subject and subject-work) supported by prompts aimed at stimulating reflexivity (106). One's own experience is seen as the result of the permanent intertwining of personal history with the social and organizational context. 2) Learning based on real problems, linked to professional practice, and shared within the training group. The goal is to develop this learning (for example, developing new professional skills and developing new practices to address shared issues during the course); it is therefore essential to identify defined and shared work themes. 3) Learning meant not only as a cognitive process, but as a process in which it is essential to consider the emotional life experiences of the subjects, whether they are related to different work-objects examined during the course or stimulated by the training context (for example, through dialogue). 4) Promoting expression and dialogue that start from representations of the self in relation to the educational problem/object. Representations (107) are considered “tools” for understanding reality (making it practicable and meaningful), but at the same time, they are often considered and established as real. It is by working on the awareness of one's own representations (for example, professional role, interlocutors) that the reflective processes stimulated during a training course can activate a process of progressive complexification of these representations in subjects. This process, if guided (by trainers and facilitators) and shared with others (participants in training groups), allows professionals to face the job market with greater awareness. Adult learning is therefore characterized as a recursive and progressive process that develops not only in a transmissive and individual way, but also through constant shared reflection (with the instructor and other professionals) on one's own experiences and personal practices. Knowledge, therefore, is not transmitted by the expert with a top-down approach, but participants are invited to share and “put into words” their habits and practices as sports coaches. These habits, regardless of the professional field of application, are usually strongly crystallized in the daily routine of professionals. The possibility of sharing and discussing these practices with a larger group allows for analysis and, by comparing them with other professional practices, supports individual and collective processes of change (5, 103). We also believe it is important to emphasize that, in line with the adopted training approach, we conducted a dialogic evaluation (108), aimed at investigating the training experience, both in terms of process and results. We believed that the training path opened new scenarios, making the practices more effective and more humanizing, also taking into account the employability of the coaches themselves.

The training perspective we promote also proposes an approach to the development of employability skills based solely on sport. To explain what we mean by this approach, we refer to the categorization of sports interventions proposed by Coalter (26) discussed earlier in the section dedicated to the state of art in this contribution. The SBS4MED training course embraces the principles of *sport-based* programs (ibid.), in which sport is conceived as a catalyst for employability skills without the need to add extra activities to develop such abilities, as would be the case in so-called *sport-plus* programs. In the SBSMED training, an alternative approach to the mainstream *de-sportivisation* of sports interventions (Ibid.) was proposed, arguing that in order to promote social outcomes through sport, it is possible to use sport without de-structuring or adapting it with the addition of extra activities. Through this approach, employability skills are inductively enhanced by normal sports training and emerge from everyday practice and the quality of the interpersonal relationship between coach and athlete, rather than being forcibly introduced through *ad hoc* exercises. Moreover, this approach does not change the way coaches carry out their training activities, but makes coaches reflect on the training methods proposed to their athletes, improving their awareness of the use of sport during daily practice.

If we consider and use sport as a useful tool to achieve an end, it is deontological to find its value within, otherwise its very assumption is lost. The engagement of people who practice a sport is based on clear, if sometimes implicit, assumptions: the pleasure of practicing a specific sport, the improvement in performance associate with it, and the desire to win are the motivational levers which truly allow learning within and through the sport context. One does not approach a sport with the intent of entering the world of work more effectively or to be included socially: these are possible consequences, not the reasons of engagement: can we imagine a concept of sport separated from that of improvement, competing and winning? The lack of effectiveness and replicability of many projects is precisely based on the involvement of young people using sport as a fictitious and temporary attraction, and then propose some other activity^3^. Sport in this perspective loses its intrinsic and authentic ability to transmit skill and develop learning, and becomes a medium, considered useful to the extent in which it is deemed attractive to involve a certain target. Our perspective, on the other hand, sees sport as a formative tool in itself, since it permits, for example, to experience continuous opportunities for relationships bases on trust, decision making, participation in conflictual and collaborative processes, the sense of self-efficacy. We see employability through the practice of pure sport, deriving from the methodology of daily training, different repertoires of skills, experienced and lived within a consciously chosen, emotionally lived, specifically taught proposal. Performance and competitiveness are therefore characteristics which are inseparable and not accessory from the idea of sport, otherwise it would mean that sport could be replaced by any other activity with the same attractive power, and it would mean believing that sport therefore, in itself, does not have a specific educational property.

This is a pillar of our perspective, which clarifies how the coach, the training methods and the concept of the game represent key issues. The more an engaging and participatory context is created, the more players will be put in a position to be active protagonists of their learning: the more situational training is promoted, the more evident il will be that one's ability to handle complex situations, assessing responsibilities, possibilities and risks is being trained, as opposed to engaging in analytical training, which tends to fractionate and reduce the complexity of the practice to single actions. If this is our interpretation of the potential of sport, the distinction between social and elite sport in some respects is nullified; knowingly living the idea that sport contexts facilitate the learning process, deriving from the sport itself self-efficacy and specific skills, is also valuable for those who practice elite sports, and who, in most cases, have made sport the focus of their lives. At the end of their career, they will be able to consciously transfer their skills to the labour market. The same applies to coaches, adopting this perspective means looking at sport as an opportunity to reflect on their practices, deriving learnings that are also useful in other contexts. In summary, the programmes that consider sport in this sense, make it a more profound and conscious practice, capable of generating individual, group and cultural learning and change, both for elite and social sports, regardless of the role played in the specific project or by peculiar needs.

Another methodological assumption underlying the SBS4MED training course is that participants have direct experience and prior knowledge of the subject matter. In traditional training, when knowledge is transmitted by an expert, participants' backgrounds and expertise remain generally implicit. In each module, participants are invited to discuss their routines as sports coaches. These routines, regardless of their professional field of application, are usually strongly crystallized in the daily lives of professionals. The opportunity to discuss these settled practices with a larger group allows them to be analyzed and, through comparison with other professionals, to support individual change processes (109). This way of implementing the training course allows for the activation of reflections around certain established routines and supports the promotion of self-awareness as professionals (60, 101). Each module of the training has been structured with the aim of fostering a debate among coaches who are constantly asked to discuss certain topics under the supervision of trainers. Through this approach, knowledge is not mechanically transmitted by the trainer, but is co-constructed with the participation of the group. New learning is thus achieved through sharing and reflection among the various participants (Ibid.). This participatory approach supports the development of social knowledge (110). The presence of the group, therefore, becomes a source for developing new learning.

### Programme overview

Providing an overview of the complete program is functional to understanding at what phase of the overall process lies the development and implementation of the training proposal that will be described below. This phase is placed in the central part of the program, which is dedicated to the planning and implementation of a training proposal aimed at coaches (WP2-3 PLANNING AND IMPLEMENTATION OF A TRAINING FOR SPORTS COACHES). Therefore, only the overall information useful for a holistic understanding of the program will be provided (Table 1).

**Table 1:** Overview of the SBSMED programme.

Work packages for the programme	Aims of the WP	Sample	Tools	Analysis of data
**WP1: RESEARCH GAP ANALYSIS**	(1) Explore what skills can be promoted by different specific sports. (2) To explore what skills are required by the labour market, according to the specific type of work. (3) To understand the gap between the skills of sport and the ones required by the labour market. (4) To provide a scientific base for structing a training for sport coaches.	**Total number of participants: 98**	Focus groups. (7 Sports-field; 6 Company corporate-business field; total number of focus groups: **13**)	Bottom-up thematic analysis, using a grounded theory perspective.
		**Sports-field: 53** (Coaches; sport managers; former professional athletes; professional athlete; sports educator; physical education teacher).		
		**Company corporate-business field: 45** (University professor; customer-marketing; intelligence specialist; HR manager; general manager of a social cooperative; recruiter; CEO Firm consultant).		
**WP2-3 PLANNING AND IMPLEMENTATION OF A TRAINING FOR SPORTS COACHES**	(1) Promote and generate a training path for coaches and strengthen the ability of sports coaches to develop employability skills in their athletes. (2) To raise sports coaches’ awareness with respect to the usage of sport as a tool for promoting employability skills.	*Phase I) Sports coaches were involved in an Action-Resource for the design and definition of training modules (SBS4MED training course).*	Thematic meetings organised within the framework of Research-Formation.	Bottom-up thematic analysis.
		**Total number of participants: 10** These were divided into two groups (*G1, G2*). This phase was conducted only in Italy, as planned by the project.		
		*G1*: 5 coaches of teams, composed of young amateurs. (Including 2 men who coached football teams, 3 women volleyball coaches). *G2*: 5 coaches of mixed teams or teams composed entirely of migrants. (All men, football coaches).		
		*Phase II) systematization of the structure and contents of the path, realization of the training course aimed at all partners and pre-post training impact assessment.*	Training modules; Pre-post training evaluation questionnaires; Pre-post training evaluation focus groups.	Quantitative descriptive analysis (questionnaires). Bottom-up content analysis (Focus Groups).
		**Total number of participants: 65** (Divided below by country, gender and sport)		
		*Italy:* **19** sport coaches. (16 males, 3 females; all football coaches); *Croatia:***13** sport coaches. (7 males, 6 females; field hockey, fencing, water polo); *Cyprus:* **10** sport coaches. (6 males, 4 females; basketball, football, handball); *Greece:* **7** sport coaches. (6 males, 1 female; all football coaches); *Portugal:* **10** sport coaches. (3 males, 7 females; swimming, pentathlon, football, futsal); *Spain:* **13** sport coaches. (All male football coaches).		
**WP4: PILOT ACTIONS AT A LOCAL LEVEL**	(1) To assess the impact and the consequences of the training at a local level. (2) To promote employability skills through local pilot actions. (3) To explore strengths and limits of sport in terms of employability.	**Participants:** *In Italy*: 3 male sports coaches, among those who participated in the training modules, with their teams. This pilot action was conducted by at least one coach and his team in each project partner country.	Semi-structured interviews, conducted before and after implementing the pilot action, to test the increase in coaches’ awareness.	Bottom-up thematic analysis.

Given the need to structure a complex project with multiple sequential objectives and the need to manage and monitor its outcomes over an extended period of time, the work was organised into four work packages (WP). Work packages are units of work, each having specific objectives, to allow the programme to be deconstructed into smaller parts, but at the same time remain cohesive and coherent, to develop related and logically designed activities within the project.

The first package, WP1: RESEARCH GAP ANALYSIS, incorporated four main objectives, 1) to explore which skills can be promoted by various specific sports, 2) to explore which skills are required by the labour market, based on the specific type of work, 3) to understand the gap between sport skills and the skills required by the labour market, 4) to provide a scientific basis for structuring training for sport coaches. The main purpose of this phase was to provide a fact-finding foundation of the skills required by the labour market, taking into consideration different occupational profiles, to understand which sport is better suited to develop those specific skills. The research questions that guided this phase were: Which skills are required by the labour market? For which specific occupational profile are these skills required? Is there a sport better suited to develop those specific skills?

This phase showed that sport is seen as a powerful vehicle for the development of employability skills: the link between specific sports and employability, however, still appeared vague and idealistic. The participants reported that it is unclear how skills developed through sport practice can be transferred to the labour market. According to the participants, one possible way to promote transferability is to work on self-awareness and the awareness of the athlete. Coaches are named as key figures in promoting the awareness of athletes. Nonetheless, the participants found it difficult to explain the methods and practical actions that have to be implemented on the field to promote employability skills through sport.

The significant results that emerged from the gap analysis confirmed and reinforced the theoretical assumptions that founded the organization of the training course. Work packages 2 and 3 (WP2-3 **PLANNING AND IMPLEMENTATION OF A TRAINING FOR SPORTS COACHES**) had the objective of (1) promoting and generating a training path for coaches and strengthen their ability to develop employability skills in their athletes; (2) raise awareness among sport coaches on the use of sport as a tool to promote employability skills. The first phase focused on the involvement of ten sport coaches in an Action-Research to design and define the topics of the training modules (SBS4MED training course). This phase, in conformity with the project, was only conducted in Italy; when the topics of the training course were designed, we moved on to phase II) which focused on improving the structure and the contents of the course. Following the fine-tuning of the course, the latter was implemented in all the partner countries involved in the project. Phase III), which focused on the assessment of the impact of the training course through a quantitative and qualitative survey, was then carried out, to examine in depth the results of the training experience.

The last work package was dedicated to conducting pilot actions at local level (**WP4: PILOT ACTIONS AT A LOCAL LEVEL**); this phase objectives were: 1) to assess the impact and the consequences of the training at a local level, 2) to promote employability skills through local pilot actions, 3) to explore strengths and limits of sport in terms of employability. In this phase, the coaches that attended the training course put into practice with their teams what they had learned. Interviews were conducted before and after the pilot phases at local level, which were analyses thematically through bottom-up classification. It showed that the training course developed a new awareness in coaches, did not subvert their daily routines, but gave them, in retrospect, food for thought on customary practices. The recognition and the transferability strategies of skills developed during the pilot action was implemented by coaches through the verbal reinforcement of the new skills learned during the training in a discussion with the athletes and the professionals in charge of the training.

## Training course: development of the content of the training modules

### Data gathering and participants

#### Action research with coaches to develop training modules (October 2020–April 2021)

During the gap analysis (WP1), it was found that research participants struggled to indicate concrete methods and actions to develop the awareness of athletes and sports coaches regarding the acquisition of employability skills through sport. What emerged is that there is a gap between the theory and practice of developing employability skills through sport. To overcome this gap, 10 thematic meetings were organised with two groups of sports coaches, to identify tools, strategies, contents and concrete methods useful for structuring training modules. These meetings constituted a transition phase from WP1 to WP2-3. The two groups consisted of 10 coaches; the first group, (G1), consisted of 5, males and females, sports coaches of teams or groups of young Italians who practised volleyball and football at amateur and professional level; the second (G2) consisted of 4 sports coaches of teams composed of migrants or migrants and Italians who practised football at amateur level. The first 6 meetings were held with group 1 (G1), the next 4 with group 2 (G2). A reflexive-participative methodology was then applied between trainers and coaches (60, 100, 101), with the aim of developing open didactic materials for the promotion of professional skills through sport (WP2-3). The aim was therefore to design with coaches (outcome), training modules for (output) coaches.

Within an approach inspired by action-research (84), the meetings were held with the aim of clarifying *how* sports coaches can be supported in the promotion of employability skills through sport and *which* open training tools and didactic materials could help coaches transmit employability skills, promoting their transferability to the labour market for young athletes. By open didactic materials we mean the continuous possibility of updating and revising the materials and tools identified, in order to emphasise, right from the design phase, the logic of circularity and progressivity that should animate this training, in which, design and practice in the field, continually question and refer back to each other, always considering partial and revisable theoretical and practical hypotheses tested. The training materials were also archived by the trainers on a web platform, accessible to participants via login credentials. The platform was used as a repository of the training materials, on the one hand to promote their continuous updating and enrichment, and on the other, to allow access to the contents also in distance mode. In fact, the training has been planned to be delivered both in presence and online according to the COVID-19 restrictions at national level. Access to the platform allows coaches to have the outline of the modules in detail (objectives, tools, methodological suggestions) and the training materials (videos, pictures, interviews), as well as some suggestions on how to use these materials.

#### Proposed themes and issues emerging from the meetings

The staff of expert trainers from the Catholic University facilitated the participation in the meetings and guided the participants in reflecting on the different topics proposed. The first six meetings were held with the first group (G1).

During the *first* meeting, the coaches were asked to reflect on *whether* and *how* sport can be used as a tool for the development of employability.

Participants reported that it is difficult for sports coaches to spontaneously focus on employability skills during training, reporting concerns about the possible transferability of sports skills to other contexts. They also suggested that educational materials should focus on the identity and day-to-day practices of sports coaches, rather than on the transferability of skills, which, according to them, is a secondary consequence of the work.

In the *second* meeting, participants were asked to reflect on how sports coaches can be guided towards a more conscious use of sport.

Participants reported that many sports coaches provide training without previous planning; they also reflected on the large gap between the management of professional and amateur sports training. This consideration led to the topic of "being professional" in amateur contexts. The coaches agreed on the importance of strengthening and developing the professional role and identity of coaches as a crucial step to guide them in a more conscious use of sport as a tool for employability. Open training materials, therefore, should focus on this key question “what does it mean to be a sports coach?”.

The *third* meeting focused on reflecting on specific methodologies that sports coaches should implement in order to make better use of sport as a channel for the promotion of employability skills.

Participants agreed that sports coaches cannot add new activities to their daily training with athletes (e.g., workshops or similar); they reported that it is important for them to have an educational figure to guide them in reinforcing what they already do on the field, without requiring them to put more effort into other activities. They agreed that didactic materials for coaches should focus on the implicit competences that sports coaches already, implicitly, transmit to their athletes; materials, therefore, should focus on the self-awareness of sports coaches, on their silent work that latently generates the development of employability competences. They also believe that this strategy constitutes a paradigm shift, as coaches are generally asked to add specific activities, including non-sporting activities, in competence development programmes through sport. According to them, the use of extra activities such as delivery employability workshops goes beyond the boundaries of sport and is something they are not really interested in managing. The participants also provided suggestions regarding training material to guide the awareness of sports coaches such as: case analyses, video analyses, exercise creation, role-plays.

The *fourth* and *fifth* meetings then focused on implicit and silent strategies implemented by coaches to develop employability skills. The trainers structured a reflective exercise to support the coaches' understanding of their own practices and how these, implicitly, develop certain employability skills.

To this end, coaches were asked to structure *football exercises* in the fourth meeting and *volleyball exercises* in the fifth meeting for their athletes from a type of training they typically practice. Afterwards, staff and coaches analysed together how much and how these "normal" exercises, i.e., not specifically designed to develop skills, were actually useful for developing certain employability skills.

Trainers and coaches reflected on how and to what extent these "normal" exercises were actually useful for developing certain employability skills. Participants made explicit the connection between their regular training and the development of employability skills such as complexity management, interpersonal skills and decision-making. These encounters were used as a pilot activity test of the training modules and also tested in WP3.

The *sixth* meeting was dedicated to sharing the findings and validating the key contents extracted from the work done together by the participants.

The results of the design were discussed with the participants, specifically on how to use them to create specific training materials for a target audience of volleyball and football coaches who train young adults at the amateur level.

The following four meetings involved the second group (G2). The staff of Catholic University guided the coaches' discussion around the delivery of sports training courses aimed at migrants.

During the *seventh* meeting, the reflection focused on the main challenges and peculiarities of working with migrants in the sports context.

Participants reported that the main challenges encountered during their career are linguistic barriers and cultural differences in the game. Specifically, the representation of football by migrants often generates conflicts on the field. In this regard, the coaches recognize the need to be trained to develop conflict and diversity management skills. However, they agree that football can be considered a common language that overcomes linguistic barriers and cultural differences, and that this sport represents an excellent tool to attract and engage this target. In fact, they report that migrants often have the expectation and desire to become professional football players, and the complex challenge linked to employability consists in guiding them in planning more realistic life goals.

The *eighth* meeting focused on the discussion of sports and employability issues for migrants. The coaches discussed how they can guide the development of employability skills for this specific target.

The coaches reported four main skills that can be improved through sports: communication, self-efficacy and self-esteem, punctuality, and empathy. Empathy, punctuality, and communication were cited as key competencies for developing integration resources and cultural intelligence. The participants also reiterated the key relational role of the sports coach, which is essential in guiding migrant athletes in acquiring these competencies. Similarly to Group 1 (G1), the importance of working on the role and identity of coaches was recurrently emphasized.

The *ninth* and *tenth* meetings focused on the implicit and silent strategies implemented by coaches to develop employability skills. The trainers structured a reflective exercise to support coaches' understanding of their own practices and how these implicitly develop certain employability skills. In this regard, the coaches were asked to structure *football exercises* in the fourth and fifth meetings, starting from a type of training that they typically practice. Subsequently, the staff and coaches analyzed together to what extent and how these “normal” exercises, i.e., not specifically designed to develop skills, were actually useful for developing certain employability skills.

The participants explicitly highlighted the connection between their habitual training and the development of specific and generic competencies that they consider useful for employability.

At the conclusion of the tenth meeting, the results were discussed with the participants in order to create specific training materials aimed at a target audience of football coaches who work with young adult migrants at the amateur level.

### Data analysis

The ten thematic meetings were recorded and transcribed verbatim by a trainer from the Catholic University. The transcriptions were analyzed thematically by two researchers from the same university, using the procedure outlined by Braun and Clarke (111). The researchers conducted an initial open and independent analysis, and then compared and discussed their results to arrive at a joint synthesis. The themes emerged through six phases of inductive analysis (111), ensuring authenticity. The first phase involved becoming familiar with the data collected by transcribing the recordings, reading them multiple times, and underlining relevant portions of the meetings. Next, the first codes were generated, with each code corresponding to a different portion of the transcript. Following an increasing abstraction process, the codes were grouped into potential main themes. The independence and coherence of each main theme were evaluated, ensuring that each theme was exhaustive and coherent with the other main themes, and similar themes were grouped together. At this point, a name was assigned to each thematic category. The final report presents five main thematic categories that became the focus of the five training modules. It is not possible to determine the degree of subjectivity and interpretation involved in the analysis, but it is considered as enriching the process of shared development of training content between trainers and coaches. The themes emerging from the report were discussed among colleagues and validated by participants through feedback meetings.

### Results: fine-tuning the training course

We consider the training modules to be a non-conclusive, simple, but technically valid tool for all institutions and individuals interested in developing discussion and sharing among coaches on the following topics: their representation of sport (Module 1), the representation of their professional coaching role (Module 2), the links between sport and employability skills (Module 3), how to concretely develop employability skills through sport (Module 4), and how to adapt sports activities based on the needs and diversity of athletes (Module 5). A summary of the training modules is proposed (Table 2: Training modules) focusing on the *themes*, *objectives*, and *tools* to implement the training proposal. Subsequently, the *content* and *underlying hypotheses* that guided the structuring of each module are described in more depth.

**Table 2:** Training modules. Themes, objectives, tools.

TRAINING CONTENTS	MAIN OBJECTIVES	DEVELOPMENT OF THE MODULE
**MODULE 1: INTRODUCTION TO SPORT** This module aims at helping sport coaches in becoming more conscious with respect to what sport means to them. The module has the purpose to guide them in understanding that their latent representation of sport impact on how they behave and use sport in their daily routine for promoting employability skills.	To co-build a shared representation of the meaning of sport	**Tool**—***What does sport mean to you?*** During the training, sport coaches are individually asked to answer the question “What does sport mean to you” by choosing an image that reflect their representation of sport. The images and the related explanation of the choice are shared among the group with trainers guiding the discussion
**MODULE 2: THE ROLE OF SPORT COACHES** The module provides coaches with a reflections and debate about their role and identity. The debate developed within this module aims to open up coaches’ minds about their identity and reinforce their consciousness about their key role as guide and mentors for their athletes.	To develop participants’ awareness about their role as sports coaches	**Tool**—“**You as a sport coach”** Sport coaches are asked to answer to the following questions on a white paper: a) What kind of coach are you? b) Which coach would you like to be? 3) Which coach should you be?; Participants share their answers and trainers encourage discussion around the three images emerged of sport coaches: the real, the desired and the ideal. **The role of the coaches:** The trainer is called to show 2–3 videos from the platform. Coaches are called upon to reflect on this key question: *What do these videos tell us about the coaches and their role? How do these coaching styles may influence the development of employability skills through sports?* The trainer guides the discussion and leads the group to reflect on the diverse representation of sport coaches emerging from the group
**MODULE 3—SPORT FOR EMPLOYABILITY SKILLS** The module aims to reinforce the practical implication of sport toward the development of employability skills.	To make participants reflect on the skills for the world of work that can be developed through sport. To develop participants’ awareness around the links between sport and employability	**Tool—Employability***–> What does employability mean to you?* Sport coaches are asked to report words that, according to them, are linked to the concept of “employability” in contemporary society. Participants and trainers discuss together the various words. **Tool—Sport and employability** à sport coaches are individually asked to write down the employability skills that can be developed through their sport. Participants share their skills and discuss convergences and divergences with the support of the trainers.
**MODULE 4: FROM THE ROLE TO THE PRACTICE** During the module, sport coaches will be guided into reflecting on their training routines as sport coaches. Starting from *what coaches YET do on the field* with the athletes, they will be asked to reflect about what employability skills they silently develop within their practice. This activity will permit to develop coaches’ awareness about the implicit skills they develop through their trainings. Then, they will be asked to structure and define practical strategy to make more explicit these skills.	To make participants think about diverse practices for developing employability skills through sport. To co-build with participants practical exercises and strategies to develop employability skills through sport	**Tool—Daily routine and practice of training**; coaches are asked to bring back an example of a training they usually implement with their athletes. From that training, they are asked to reflect on the employability skills they have developed. This exercise is powerfully useful to make more explicit certain skills that sport coaches already develop in their athletes without awareness. **Tool—Inspiring coaches** (link with the platform; in the web-platform it is possible to find diverse videos and written sport exercises; it's up to trainer which one to show during the training)→ These videos can be used as a tool for promoting critical think in sport coaches. The scope of this exercise is to develop sport coaches’ capacity to observe, critique and change their own actions. **Tool**—**Role playing** (link with football and volleyball exercises on the web platform; the tool can be used if the training is in presence and there is adequate space)
**MODULE 5: DIVERSITY MANAGEMENT** The module is fundamental to adapt and contextualize the development of employability skills through sport considering the specific athletes’ needs and differences at a local level.	To promote reflection on the concept of difference and diversity within sport. To promote reflection related to how to manage diversity and how to adapt interventions coherently with athletes’ needs and characteristics (gender, culture, individual characteristics, ethnicity, religion or social / economic status)	**Tool**—**Diversity and differences** (link with videos in the web-platform)—The trainer can show in the classroom 1–2 video among those present on the platform. Starting from these videos, participants are asked to answer to these questions: What are the main characteristics of your team in terms of diversity? What is diversity for you? To which diversity category (gender, culture, individual characteristics, ethnicity, religion or social / economic status, sport skills level) do you mostly pay attention during your training? The trainers collect the answers and guide the group to converge towards a shared definition of diversity. **Tool—Diversity in your team**: in this section, sport coaches are asked to answer to this question “What is the most prevalent diversity in your team? Choose among the type of diversity here proposed: age diversity, race diversity, gender diversity, personality diversity, sport skills diversity, religion or social / economic status diversity”. This tool is functional to highlight the specificity of each sport team and help sport coaches to develop awareness with respect to the unique characteristics of their team. **Tool—Diversity and difference** (link with slide presentation in the platform) → This slide presentation shows a definition of “diversity” and “difference” and propose some practical strategies to manage diversity in sport.

### Contents and underlying hypotheses

#### Module 1: sport in general and the sport that I am coaching

The training contents proposed in Module 1 aim *to increase the awareness of sports coaches about what sport means to them*. The module aims to bring to light their latent representation of sport, which influences their daily actions, to make it more articulated and shared. During the training, sports coaches are individually asked to answer the question “*What does sport mean to you?”* by choosing an image that reflects their representation of sport. The selected images, and their meaning, are shared within the group, while the trainers facilitate the discussion. The underlying hypothesis is that many coaches have an implicit and not fully articulated representation of sport, even rigorous in some cases, derived mainly from their own experience. It seems necessary, therefore, to promote a multidimensional view of the world in which they operate, to avoid trivializing its peculiar processes.

#### Module 2: the coach

Module 2 facilitates a reflection on the coach's identity as a facilitator of the athletes' learning process. The specific objective, therefore, concerns *the development of awareness of one's own style and the possibility of accessing other ways of interpreting one's role depending on the interlocutor and the objective*. Coaches are invited to answer three questions: (1) What type of coach are you?, (2) What type of coach would you like to be?, (3) What type of coach should you be? Participants share their answers, and trainers encourage thoughts and reflections starting from the comparison between the three emerging representations: the real, the desired and the ideal. As a support tool designed to achieve the same objective, trainers show two or three videos available by accessing the training materials repository. Coaches are asked to watch them after the following two questions have been asked: (1) What can these videos tell us about coaches and their role? (2) How can these coaching styles influence the development of crucial skills for this sport and useful for work (imagining specific jobs)? The trainer leads the discussion and supports the group as they reflect on the different representation of sports coaches emerging from the group.

The assumption is that rigid role codes do not allow for flexibility based on situations (age, history, goals, etc.) and create rather intransigent training practices and methods, with little room for innovation and creativity. Being able to recognize and access different emotional ranges allows better access to different relationships with young athletes.

#### Module 3: Job market and employability skills

Module 3 invites coaches to identify the skills considered crucial in the job market, particularly in specific occupations. For example, they are asked to present some words related to the concept of “employability” in contemporary society. Subsequently, participants and trainers discuss the proposed words and compare them with data on employability in different fields and the characteristics of the current job market (which ideally are updated regularly). A second phase sees participants sharing the skills they have in mind with the group, followed by a discussion on points of agreement and disagreement in their proposals. The aim is to increase knowledge and deepen some aspects related to the theme of work and employability.

#### Module 4: training skills

In Module 4, coaches are specifically asked to work on their coaching practices. Starting from their practices on the field with athletes, they are asked to reflect on what specific skills are necessary for good sports performance and about methodology they use. Subsequently, the identified repertoire is compared with the needs of the job market. This activity promotes the development of greater awareness of the skills that can be acquired through training and competition. In addition to stimulating reflection and creativity in delineating the repertoire of practices, coaches are required to define a practical strategy to make these skills more explicit, more recognizable. The specific objective of this training module is to improve existing exercises and strategies or devise new ones during the exchange between participants, to develop skills related to sports performance that can also be potentially useful in the labor market. They are then asked to share with the group a typical training method they use with their athletes. With this approach, employability skills are inductively derived from normal sports training, emerging from the daily activities proposed by the coach, rather than being forcibly introduced through an *ad hoc* exercise. The goal, therefore, is not to change the way coaches train athletes, but to invite coaches to reflect retrospectively on the methods proposed to improve performance and their awareness of the potential resources of sport when practiced according to coherent methods. An important basic assumption is that it is necessary to examine the merits of training practices. The idea is that some training methods may better promote a player's growth, facilitating the development of specific sport skills and at the same time important components in terms of employability. For example, let's consider different training methods: imagine a type of training with a predominance of analytical exercises, with many repetitions, the individual athlete training against a wall, training without an opponent, or codified training practices to be followed. Instead, let's imagine situational training sessions, with greater complexity of variables to consider, interpretive discretion, the need for rapid decisions, interaction between players, options. It is easy to understand which alternative will be more useful for developing cooperation and speed in the decision-making process, considered two fundamental qualities in football and other professions. Alternative training methods to those based on repetitive and mechanical actions are desirable, methods that oppose the culture that sees errors as mistakes to avoid and punish, that do not continuously break the game into individual athletic gestures, but rather seek to recreate situations and training sessions based on relationships. Training sessions that see players confront the proposed methodology and activate peer learning processes (through video analysis, experimentation of training methods proposed by the players themselves, sharing of objectives, and joint verification) appear effective.

#### Module 5: diversity management

In Module 5, coaches are encouraged to reflect on the importance of contextualizing and adapting each training proposal and session to the employability skills, specific needs, and differences of athletes. A reflection on the concepts of diversity and difference in sports is thus encouraged, considering the management practices of interventions, to adapt them to the specific needs and peculiar characteristics of athletes (e.g., gender, individual characteristics, culture, religion, social and economic status). The instructor can then show one or two videos in the classroom (also available on the web platform), asking participants to respond to these questions: What does diversity mean to you? What are the main characteristics of your team in terms of diversity? Which category of diversity (individual characteristics, gender, culture, level of sports skills, etc.) do you pay the most attention to during your training? The instructors then collect the answers and guide the group towards a shared definition of diversity, offering practical strategies and suggestions for managing diversity in emerging local contexts.

This tool is considered functional in highlighting the specificity of each sports team and helping sports coaches develop awareness of the unique characteristics of their players and their team.

## Discussion

We believe that training can be a valuable tool to foster the emplyability of young people through sport. For this reason, after having identified some theoretical and methodological weaknesses, in many of the training programmes proposed in the literature, we have tried to challenge these critical issues in the SBSMED training programme.

The target group of the SBSMED experience are coaches, who are mainly considered the "great absentees" of training projects aimed at developing skills to increase employability through sport. Stimulating coaches' awareness of their role and training methodologies, the importance of generating trusting relationships with their athletes through good sporting practice, appears to be a fundamental resource for supporting authentic employability. This overcomes the idea that employability is reduced to competences understood as objects, automatically transferable to the work environment, or, on the contrary, that competences can only be developed by adding content to sport, which deforms its nature.

Within an action-research logic, the training was set up as a dialogical process concerning the participants' professional experience (5, 112, 113). The opportunity to reflect together on their practices made it possible to make them explicit and evaluable, thus supporting processes of change as individuals and as groups.

Coaches are guided through a training process that supports them in knowing how to reflect on their function, why they should choose one training methodology over another, how to recognise in their specific practices what they are training, highlighting and enhancing it. It is a matter of doing better and more consciously what one already does (good training and competition sessions, locker room management…) without resorting to additional learning devices of a non-sporting nature. In this way, employability skills are inseparable from sporting practice, the repertoires of skills are contextual and changeable, set in a solid and valuable relational framework, implicitly not borrowing the idea that the educational and social purpose is unrelated to sport.

The participants' evaluation of the training course showed strong professional and personal growth (greater clarity of the function acted, technical choices made, repertoires of skills trained, trust established and the effort and risks involved). The participants' synthesis was therefore that the programme enabled them to “*improve as coaches in terms of our concrete ability to coach*”.

This, in our opinion, is the key to a real involvement of coaches along the whole pathway. This avoids the typical dynamics ranging from resistance to engage in such programmes to strong initial impulses of idealisation, which are often followed by disinvestment.

It is clear that the SBSMED programme can therefore be considered a first step to contribute to the debate. Looking ahead, there is a need to involve athletes as well, in order to investigate the perceived and/or actual positive influence on their growth in terms of sports performance and perceived greater employability. Furthermore, a more comprehensive and in-depth impact evaluation should not stop at the involvement of teams and athletes to determine the “success of the program”, but it would be desirable to involve other stakeholders as well, to evaluate whether what the program beneficiaries have learned has a real chance to be put into practice in the job market, resulting in greater access opportunities and translating into “social success or failure” (29). The risk is implicitly assuming that employability is a private matter and an individual responsibility, legitimizing the use of an unfair guilt-inducing and classifying logic, the same one that seems to be used when defining aprioristic targets, implicitly endowed with negative connotations, such as “NEET”, “migrants”, and “young people” (21).

In conclusion, we consider it important to emphasise how necessary it is to also work with the organisational cultures of sports clubs and federations: *how can sports practice be improved so that it can truly support employability? What support and ongoing training for coaches and academy managers?*

The issue of employability should not be reduced to packages of skills, nor to a mere individual responsibility (either of the young person or of the coach). Rather, it is the work of a subject embedded in a culture of synergy, which is expressed between different actors and is aimed at generating quality conditions of interpersonal and group bonds, of competent practices, of connections with the various stakeholders in the world of sport, school, work and new social cultures (5, 62, 114). We believed that the training course opened up new scenarios, which made the practices more effective and more humanising, also taking into account the employability of the coaches themselves as well as the athletes, thus opening up to a circular, processual, relational and virtuous idea of employability.

## Footnotes

^1^Appendix section contains a description of the research process and results preliminary to the outlined critical-thematic synthesis.

^2^ https://asag.unicatt.it/asag-ricerca-e-progetti-skills-by-sport-for-med-sport-as-a-vehicle-for-developing-skills-for-the-labor

^3^These considerations are derived from a ten-year collaboration with the AC Milan youth sector, the AC Milan football school network (amateurs) and the Ac Parma youth sector.

## Appendix

The elements identified in the text as *critical* and which characterise the subject of training, promoted and implemented in projects and programmes that aim to increase employability through sport, are the result of a reasoned synthesis. In fact, we interrogated the existing literature through various searches, both extensive (Tables 3, 4) and more specific (Tables 5, 6), which returned sparse and fragmented results. For the sake of completeness, we report the results of the research carried out prior to identifying the critical recurrences and shortcomings that seem to qualify the state of the art, so that it is possible to observe the transparent research process that led to the present statements. From the analysis carried out, there appears to be a lack of studies capable of providing a broad, explicit and in-depth overview of the use of training for the strategic purposes of increasing or supporting employability through sport.

**Table 3:** SfE literature review.

Database	Research type	Query	*N* Review	Review
APA PsychINFO		tiab (Employability) AND tiab (sport) AND tiab (review)	1	Arvinen-Barrow, M. (2010). Review of Applied sport psychology: A case-based approach. The Sport Psychologist, 24 (1), 117–118. https://www.proquest.com/scholarly-journals/review-i-applied-sport-psychology-case-based/docview/622251504/se-2
Google Scholar		"sport for employability"	0	
	Limited to: Review article	"employability through sport"	0	
		Allintitle: employability sport	1	Ng, G. X. T. (2016). A systematic review: sport management undergraduates’ employability.
Elsevier Scopus		TITLE-ABS-KEY (“Employability through sport”)	0	
		TITLE-ABS-KEY (“sport for Employability”)	0	
	Limited to: “article” and “review”	TITLE-ABS-KEY (“employability AND sport AND review)	7	Budzynski-Seymour, E., Conway, R., Wade, M., Lucas, A., Jones, M., Mann, S., & Steele, J. (2020). Physical activity, mental and personal well-being, social isolation, and perceptions of academic attainment and employability in university students: the Scottish and British Active Students Surveys. Journal of Physical Activity and Health, 17 (6), 610–620.
				Coalter, F., Theeboom, M., & Truyens, J. (2020). Developing a programme theory for sport and employability programmes for NEETs. International journal of sport policy and politics, 12 (4), 679–697.
				De Schepper, J., & Sotiriadou, P. (2018). A framework for critical reflection in sport management education and graduate employability. Annals of Leisure Research, 21 (2), 227–245.
				Dinning, T. (2017). Preparing sports graduates for employment: Satisfying employers’ expectations. Higher Education, Skills and Work-Based Learning.
				Foster, S. B., & Pierce, D. A. (2021). Improving experiential learning in sport management through work-integrated learning. Sport Management Education Journal, 15 (2), 117–126.
				Miragaia, D. A., & Soares, J. A. (2017). Higher education in sport management: A systematic review of research topics and trends. Journal of Hospitality, Leisure, Sport & Tourism Education, 21, 101–116.
				Van Deyk, K., Moons, P., Gewillig, M., & Budts, W. (2004). Educational and behavioral issues in transitioning from pediatric cardiology to adult-centered health care. Nursing Clinics, 39 (4), 755–768.
SAGE journal		Title (sport AND employability AND Review)	0	
		Abstract (sport AND employability AND Review)	0	

**Table 4:** SfE scientific papers.

Database	Research limits	Query	*N* Article	Article
Elsevier Scopus	Limited to “journal”	TITLE-ABS-KEY (“Employability through sport”)	0	
	Limited to “article”	TITLE (employability AND sport)	17	Ahmad M.F., Safwan N.S.Z., Dahlan N.D., Bakri N.H.S., Aznan E.A.M., Tumijan W. (2022) Exploring Human Resource Management Practices and Employability: A Study on Sports Graduates in Malaysia, *Frontiers in Sports and Active Living*, 4, 1001435, 10.3389/fspor.2022.1001435
				Burnett C. (2023) Sport-for-employability as an innovative practice in addressing youth underemployment in sub-Saharan Africa, *Frontiers in Sports and Active Living*, 4, 942479, 10.3389/fspor.2022.942479
				Burnett C. (2022) Employability pathways in a sport-for-development programme for girls in a Sub-Saharan impoverished setting, *Journal of Physical Education and Sport*, 22 (4), 109, 863–869, 10.7752/jpes.2022.04109
				Coalter F., Theeboom M., Truyens J. (2020) Developing a programme theory for sport and employability programmes for NEETs, *Sport, Business and Management: An International Journal*, 12 (2), 117–134, 10.1108/SBM-02-2021-0013
				Commers T., Theeboom M., Coalter F. (2022) Exploring the design of a sport for employability program: A case study, *Hong Kong journal of Social Sciences*, 59, 112–124
				de Schepper J., Sotiriadou P. (2018) A framework for critical reflection in sport management education and graduate employability, *Journal of Hospitality, Leisure, Sport and Tourism Education*, 28, 100306 10.1016/j.jhlste.2021.100306
				de Subijana C.L, Ramos J., Garcia C., Chamorro J.L. (2020) The employability process of Spanish retired elite athletes: Gender and sport success comparison, *Cultura, Ciencia y Deporte,* 16 (4), 7, 39–48, 10.12800/CCD.V16I47.1694
				Dinning T. (2017) Embedding employability and enterprise skills in sport degrees through a focused work—based project a student and employer viewpoint, *European Sport Management Quarterly* 21 (2), 280–301, 10.1080/16184742.2020.1742184
				Finch D.J., O'Reilly N., Legg D., Levallet N., Fody E. (2022) So you want to work in sports? An exploratory study of sport business employability, *International Journal of Sport Policy and Politics* 12 (4), 679–697 10.1080/19406940.2020.1832136
				Lord R., Lorimer R., Babraj J., Richardson A. (2019) The role of mock job interviews in enhancing sport students’ employability skills: An example from the *UK Sport, Education and Society*, 24 (8), 883–903, 10.1080/13573322.2018.1482265
				Lu H.-F. (2021) Enhancing university student employability through practical experiential learning in the sport industry: An industry-academia cooperation case from Taiwan, *Education and Training*, 60 (5) 458–472, 10.1108/ET-11-2017-0179
				Martin L., West J., Bill K. (2008) Incorporating problem-based learning strategies to develop learner autonomy and employability skills in sports science undergraduates, *Annals of Leisure Research* 21 (2), 227–245, 10.1080/11745398.2017.1336107
				Minten S. (2010) Use them or lose them: A study of the employability of sport graduates through their transition into the sport workplace, *International Journal of Sport Policy*, 9 (3), 431–451, 10.1080/19406940.2017.1359648
				Minten S., Forsyth J. (2014) The careers of sports graduates: Implications for employability strategies in higher education sports courses, *Journal of Hospitality, Leisure, Sport and Tourism Education,* 20 1–9, 10.1016/j.jhlste.2016.11.002
				Raven S. (2018) Mind the gap: Sport management education and employability auto-ethnographical analysis of sport management education and the sports fitness industry, *Journal of Hospitality, Leisure, Sport and Tourism Education*, 15 (1), 94–102, 10.1016/j.jhlste.2014.06.004
				Smismans, S., Wylleman, P., De Brandt, K., Simon, D., Vitali, F., Ramis, Y., Torregrossa, M., Lobinger, B., Stambulova, N. B., & Cecić Erpič, S. (2021). From elite sport to the job market: Development and initial validation of the Athlete Competency Questionnaire for Employability (ACQE) [Del deporte de élite al mercado laboral: Desarrollo y validación inicial del Cuestionario de Competencias de Deportistas para la Empleabilidad (ACQE)]. Cultura, Ciencia Y Deporte, 16 (47), 39–48. doi: 10.12800/ccd.v16i47.1694
				Spaaij R., Magee J., Jeanes R. (2013) Urban Youth, Worklessness and Sport: A Comparison of Sports-based Employability Programmes in Rotterdam and Stoke-on-Trent, *Motricidade*, 8 (4), 26–37, 10.6063/motricidade.8 (4).1549
Google Scholar	with the exact phrase	Allintitle: “sport for employability"	5	Commers, T., & Theeboom, M. (2021, September). Exploring claims of Sport for Employability programmes: An assist to the job market? In 17th European Association for Sociology of Sport Conference:“Sports in the face of the global health crisis of COVID 19. Great social challenges” (p. 53). European Association for Sociology of Sport.
				Commers, T., Theeboom, M., & Coalter, F. (2019). Exploring claims of sport for employability programmes: A case study of a sport for employability programme located in Flanders. In Abstract Book EASS (pp. 111–111). European Association for Sociology of Sport.
				Commers, T., Theeboom, M., & Coalter, F. (2022). Between input and impact: Key mechanisms of Sport for Employability programmes based on (ex)-participants’ perspectives. In World Congress of Sociology of Sport: 2022 EASS & ISSA (pp. 217–289). Eberhard Karls Universität Tübingen.
				Morgan, H., Bush, A., & Bowles, H. (2022). Active for Employment: Enhancing employability through sport and physical activity participation Final Report.
				Moustakas, L., Raub, V., Moufagued, Y., & Petry, K. (2022). From Sport to Work? Exploring Potentials in a Moroccan Sport-for-Employability Programme. Youth, 2 (4), 759–771.
	with the exact phrase	"employability through sport"	0	
	with the exact phrase	Allintitle: “sport employability”	4	Allen, K., Bullough, S., Cole, D., Shibli, S., & Wilson, J. (2013). The impact of engagement in sport on graduate employability. British Universities and Colleges Sport, 2013, 1–59.
				Beaumont, E. (2012, April). The Increasing students’ awareness of employability: Embedding careers education across marine sport programmes. In HEA STEM Annual Conference, Imperial College, London (pp. 12–13).
				Bellantonio, S., & Domenico, T. (2019). Sport d’élite ed employability: presupposti teorici, orientamenti politici e prospettive educative. FORM@ RE, 19 (2), 364–376.
				Bellantonio, S., & Tafuri, D. (2019). Élite sport & employability: Theoretical frameworks, policies and educational perspectives. Form@ re-Open Journal per la formazione in rete, 19 (2), 364–376.
SAGE journals	Limited to Title	Sport for employability (Same result for Sport AND employability)	2	Spaaij, R., Magee, J., & Jeanes, R. (2013). Urban Youth, Worklessness and Sport: A Comparison of Sports-based Employability Programmes in Rotterdam and Stoke-on-Trent. Urban Studies, 50 (8), 1608–1624. doi: 10.1177/0042098012465132
				Grant, M. T., Hanlon, C., & Young, J. A. (2022). An employable graduate: Essential awareness factors to the preparation of sport management practical experiences. Industry and Higher Education. doi: 10.1177/09504222221147129
APA PsycINFO + Elsevier Scopus	APA PsycINFO: Limited to “academic journal"	title(employability) AND title(sport)	4	de Schepper, J., Sotiriadou, P., & Hill, B. (2021). The role of critical reflection as an employability skill in sport management. European Sport Management Quarterly, 21 (2), 280–301. doi: 10.1080/16184742.2020.1742184
	Elsevier scopus: Limited to “article"	TITLE (employability AND sport)		Miragaia D., Carvalho P.G. (2012) Analysis of methodologies for assessing the employability of Sport graduates in Portugal [Análise das metodologias de avaliação da empregabilidade dos Graduados em Desporto de Portugal], *Cogent Education*, 4 (1), 1387085, 10.1080/2331186X.2017.1387085
				Hall E.T., Cowan D.T., Vickery W. (2019) ‘You don't need a degree to get a coaching job’: investigating the employability of sports coaching degree students, *Journal of Hospitality, Leisure, Sport and Tourism Education*, 25, 100195, 10.1016/j.jhlste.2019.04.001
				Vilanova A., Puig N. (2013) Combining a career in sports with an academic career for future employability: Is it a question of strategy? [Compaginar la carrera deportiva con la carrera académica para la futura inserción laboral: ¿una cuestión de estrategia?], *Journal of Hospitality, Leisure, Sport and Tourism Education*, 7 (1), 18–30, 10.3794/johlste.71.169
Elsevier Scopus + Google Scholar	Google Scholar: With the exact phrase	Allintitle: “sport for employability"	6	Burnett, C. (2022). Sport-for-employability as an innovative practice in addressing youth underemployment in sub-Saharan Africa. Frontiers in Sports and Active Living, 4.
	Elsevier Scopus: Limited to “journal”	TITLE-ABS-KEY (“sport for Employability”)		Coalter, F., Theeboom, M., & Truyens, J. (2020). Developing a programme theory for sport and employability programmes for NEETs. International journal of sport policy and politics, 12 (4), 679–697.
				Commers, T., Theeboom, M., & Coalter, F. (2022). Exploring the design of a sport for employability program: A case study. Frontiers in sports and active living, 4.
				Griffiths K., Bullough S., Shibli S., Wilson J. (2017) The impact of engagement in sport on graduate employability: implications for higher education policy and practice, *International Journal of Environmental Research and Public Health*, 17 (15), 5460, 1–12, 10.3390/ijerph17155460
				Tsitskari E., Goudas M., Tsalouchou E., Michalopoulou M. (2017) Employers’ expectations of the employability skills needed in the sport and recreation environment, *Managing Leisure*, 15, 44958, 67–82, 10.1080/13606710903448061
				Sato S., Kang T.-A., Daigo E., Matsuoka H., Harada M. (2021) Graduate employability and higher education’s contributions to human resource development in sport business before and after COVID-19, *Urban Studies*, 50 (8), 1608–1624, 10.1177/0042098012465132

**Table 5:** SfE and training literature review.

Database	Research limits	Query	*N* Review	Review
APA PsychINFO		tiab (Sport for Employability) AND tiab (training) AND tiab (review)	0	
Elsevier Scopus		TITLE-ABS-KEY (“Sport for employability” AND training AND review	0	Coalter, F., Theeboom, M., & Truyens, J. (2020). Developing a programme theory for sport and employability programmes for NEETs. International journal of sport policy and politics, 12 (4), 679–697.
Google Scholar	Limited to: Review article	"sport for employability” training	0	
SAGE journal		Title (sport for employability) Title (training) Title (review)	0	
		Abstract (sport for employability) Abstract (training) Abstract (review)	0	

**Table 6:** SfE and training scientific papers.

Database	Research limits	Query	N Article	Article
**APA PsychINFO**		tiab (sport for employability) AND tiab (training)	4	Vilanova, A., & Puig, N. (2013). Compaginar la carrera deportiva con la carrera académica para la futura inserción laboral: ¿Una cuestión de estrategia? [Combining a career in sports with an academic career for future employability: Is it a question of strategy?] Revista De Psicología Del Deporte, 22 (1), 61–68. https://www.proquest.com/scholarly-journals/compaginar-la-carrera-deportiva-con-académica/docview/1348794609/se-2
				Campos-Izquierdo, A., González-Rivera, M. D., & Taks, M. (2016). Multi-functionality and occupations of sport and physical activity professionals in Spain. European Sport Management Quarterly, 16 (1), 106–126. doi: 10.1080/16184742.2015.1108990
				Li, K., Peng, M. Y., Du, Z., Li, J., Yen, K., & Yu, T. (2020). Do specific pedagogies and problem-based teaching improve student employability? A cross-sectional survey of college students. Frontiers in Psychology, 11, 12. doi: 10.3389/fpsyg.2020.01099
				Li, K., Peng, M. Y., Du, Z., Li, J., Yen, K., & Yu, T. (2020). “Do specific pedagogies and problem-based teaching improve student employability? A cross-sectional survey of college students": *Corrigendum*. Frontiers in Psychology, 11, 1. doi: 10.3389/fpsyg.2020.01872
**Elsevier Scopus**		TITLE-ABS-KEY (“Sport for employability” AND training)	2	Coalter, F., Theeboom, M., & Truyens, J. (2020). Developing a programme theory for sport and employability programmes for NEETs. International Journal of Sport Policy and Politics, 12 (4), 679–697. doi: 10.1080/19406940.2020.1832136
				Commers, T., Theeboom, M., & Coalter, F. (2022). Exploring the design of a sport for employability program: A case study. Frontiers in Sports and Active Living, 4. doi: 10.3389/fspor.2022.942479
**Google Scholar**	In the title of the article	*with all of the words* training *with the exact phrase*: “sport for employability"	0	
	Anywhere in the article	*with all of the words* training *with the exact phrase*: “sport for employability	0	
**SAGE journals**	Limited to Title	Sport for employability AND training	0	
	Limited to Abstract	Sport for employability AND training	0	

The literature on Sport for Employability (SfE) is part of the broader area of Sport for Development (SfD) studies. The latter includes a large and growing collection of studies interested in how sport can be usefully applied to achieve positive developmental outcomes, stimulate relevant change, and overcome social inequalities (115, 116). The state of the art, also, shows that studies concerning sport and employability in the narrow sense are highly fragmented. In fact, such studies are reported, structured and implemented on the basis of arbitrary criteria, which makes systematisation difficult (19, 27). As evidence of such a thematic, theoretical and methodological fragmentation, there is a gap in the literature regarding reviews on the SfE theme. Indeed, studies that provide a broad and in-depth overview of the use of sport to increase or support employability seem to be absent. This lack, in our opinion, demands to be not only highlighted, but also interpreted.

To substantiate this statement, we present the outcome of a search carried out on four relevant databases (APA PsycInfo, Elsevier Scopus, Google Scholar, SAGE Journals), as a demonstration of the scarcity of studies on the specific SfE topic. Several queries have been entered through the strict use of keywords: “sport” and “employability”. Several queries were entered using only the keywords “sport” and “employability”. No time or language restrictions were imposed on the search, given the relatively recent history of the topic (67). In all databases, the search was filtered for academic and scientific articles only. When the most significant query for the present research “sport for employability” (SfE) returned results that were not numerically remarkable, the search was expanded to the query “sport and employability”. When the query “sport and employability” returned results that were too general and therefore not interesting for the specificity of the present study (violating the relevance criterion, e.g., all those studies containing the word “employability” extensively in the text), studies containing “sport and employability “only in the title” or “only in the abstract” were included. These limitations were imposed according to the margin of possibility that the named databases allowed.

The queries used did not return any reviews on the SfE topic of our interest, i.e., regarding the use of sports training to increase employability (Appendix, Table 3: SfE literature reviews). In addition, the results in terms of scientific articles appear numerically scarce, so that the full results of the research could be reported in the dedicated table (Appendix, Table 4: SfE scientific papers). Proceeding more specifically, following the same procedure as described above and entering the queries “sport for employability” and “training”, even poorer results are observed, both in relation to reviews (Table 5: SfE and Training Literature review) and to scientific articles on the subject (Table 6: SfE and Training Scientific papers). The four tables show the transparent research process that led to the present conclusions, so that their consistency can be replicated or verified. The numerical paucity and thematic fragmentation found is also confirmed by other authors in recent authoritative contributions on the subject of SfE (19), who state that they have to refer to the more general SfD literature even in studies dedicated to SfE “*given the limited number of studies on increasing employability through sport*”. This outcome, despite being partial and unsystematic (65), is reported in this manuscript not to draw conclusions, but to highlight how premature an attempt at systematisation based on the existing literature is. It should be pointed out that a systematisation would be premature not because the literature is meagre and multifaceted (66), but because it does not present specific considerations on design intentionality. Indeed, it merely describes what was carried out and not the contextual, theoretical, and methodological configurations that produced successful or unsuccessful targeted impacts (25, 117).
